# A Cross-sectional Analysis of Socio-spatial Patterning of Tobacco Retail in Shanghai, China

**DOI:** 10.1093/ntr/ntac155

**Published:** 2022-07-02

**Authors:** Chunyu Zheng, Zhiqiang Feng, Jamie Pearce

**Affiliations:** Centre for Research on Environment, Society and Health, School of GeoSciences, University of Edinburgh, Edinburgh, UK; Centre for Research on Environment, Society and Health, School of GeoSciences, University of Edinburgh, Edinburgh, UK; Scottish Centre for Administrative Data Research, University of Edinburgh, Edinburgh, UK; Centre for Research on Environment, Society and Health, School of GeoSciences, University of Edinburgh, Edinburgh, UK

## Abstract

**Introduction:**

International evidence from high-income countries demonstrates that the availability of tobacco tends to be greater in more urban and more deprived neighborhoods. However, little is known about the socio-spatial disparities in other settings, including megacities in China. This study investigated the patterning of tobacco retailers across Shanghai by types of tobacco retailers, including the relationship with levels of urbanity and neighborhood deprivation.

**Aims and Methods:**

Tobacco retailer data (*n* = 19 413) was extracted from a web-scraped Points-of-Interest database. For all communities (*n* = 5432) across Shanghai, neighborhood tobacco retail availability was calculated using population-weighted kernel density estimation and grouped by quintiles of neighborhood deprivation and a 3-level urban classification. Associations were analyzed using the Kruskal–Wallis tests and epsilon squared.

**Results:**

Across Shanghai, tobacco retail availability decreased from more urbanized areas to less urbanized areas. There was a statistical difference (*p* < .001) in the availability of tobacco retail across quintiles of deprivation, with the highest availability in the less deprived neighborhoods, and the lowest availability in the most deprived neighborhoods. However, this trend was reversed in the urban center, where retail availability was greatest in the most deprived areas. Convenience stores were the most common type of tobacco retailer across the city, while tobacco-only outlets were most strongly associated with levels of neighborhood deprivation.

**Conclusions:**

The results show an association between tobacco retail availability and neighborhood deprivation, which varied with levels of urbanity and types of tobacco retailers. These findings provide supportive evidence for further interventions that target reducing inequalities in exposure to tobacco retail.

**Implications:**

This is the first study to examine the relationship between tobacco retail availability and neighborhood deprivation in the context of Chinese megacities. Using data from Shanghai, China, we found a significant non-linear association between tobacco retail availability and neighborhood deprivation across the city. It is plausible that the socio-spatial disparities in tobacco retail availability at the neighborhood level may be a key factor explaining differences in smoking behaviors between sociodemographic groups. The findings emphasize the need for greater efforts in regulating neighborhood-level tobacco retailing in China.

## Introduction

China is the largest tobacco manufacturer and consumer worldwide, accounting for 30% of the world’s tobacco production and 40% of the tobacco consumption, with more than 300 million current smokers.^1^ In 2018, the smoking prevalence in China was 26.6% (50.5% among men and 2.1% among women).^2^ More than 1.4 million cases of premature mortality can be attributed to smoking-related diseases every year.^3^ Given the high prevalence of smoking and the tremendous burden of tobacco-related diseases, the development of successful tobacco control policies is a key public health priority in China.

In addition to individual-level factors (eg socioeconomic status,^4^ gender,^5^ mental health status^6^), previous international research has demonstrated that a number of local environmental factors (eg neighborhood disadvantage,^7,8^ local social norms,^9^ neighborhood crime,^10^ disorders and stressors^8^) explain the social and spatial differences in smoking behaviors. Tobacco retail availability has also been identified as a key environmental factor that likely contributes to tobacco consumption.^7,11,12^ Better access to tobacco retailers, such as higher density or closer residential proximity, can promote smoking by providing opportunities to obtain products, raise tobacco brand awareness, create a competitive local market that might reduce product costs, and influence social norms regarding tobacco consumption.^13^ Prior studies from North America, the United Kingdom, and New Zealand suggest an association between better tobacco retail availability and a higher chance of smoking initiation amongst adolescents,^14,15^ higher adult smoking prevalence,^11,12^ and lower odds of cessation,^12,16,17^ emphasizing the importance of regulating tobacco retail availability as part of a comprehensive tobacco control strategy.^17^

The tobacco industry strategically targets low-income, disadvantaged, and minority populations.^18^ Studies from high-income countries (HICs), including the United States,^19,20^ Canada,^21^ Australia,^22^ New Zealand,^23^ and Scotland,^24^ show that tobacco retailers tend to concentrate in more deprived,^21–24^ more urbanized,^19,24^ or high-minority neighborhoods.^19,20^ Residents living in deprived communities may have limited information about the health hazards of smoking, as well as a lack of social support and resources (eg health care, employment opportunities), which increases their susceptibility to taking up smoking^25,26^ and is likely to result in greater disparities in tobacco use and subsequent health outcomes.^26^

Although strong social gradients in tobacco retailing have been found in HICs, little research has been carried out on the socio-spatial patterning of tobacco retailing in other settings, including urban China,^27^ where tobacco retailing remains ubiquitous.^28,29^ Previous work on tobacco retailing in China, such as a study in Hangzhou,^28^ has relied on audit sampling to acquire data on tobacco retailers, which is likely to lead to incomplete and potentially biased information that underestimates the retail distribution, particularly in less urbanized areas. Another study in Changsha relying on audit sampling, found a positive but non-significant association between the total number of tobacco retailers within each neighborhood and the average property price in the neighborhood.^30^ Similarly, Wang et al. (2016) demonstrated that illicit tobacco retailers tended to be concentrated in the urban center and in high population density areas.^27^

Therefore, there is a need for a more robust evidence base examining the social patterning of tobacco retailing in China. Many of the factors driving the socio-spatial distribution of tobacco retailing in HICs have become increasingly pertinent in the Chinese context, particularly over a period of dramatic urban transformation that has fundamentally changed the community structures and social order of Chinese cities.^31^ These rapid urban changes have been accompanied by shifts in tobacco use, including the concentration of smoking amongst more disadvantaged groups.^31,32^ Further, growing inequalities in land prices and rental costs in urban China may lead to a preponderance of tobacco retailing in more deprived areas, and in turn a greater concentration of smokers in less advantaged neighborhoods.^33^ Given the rapid transformation of the economy and its urban form, we hypothesized that the socio-spatial patterning of tobacco retail in the megacity of Shanghai may share similarities with those documented in HICs.

The State Tobacco Monopoly Administration (STMA) is the governmental body that oversees the availability of tobacco in China.^34,35^ The local branch of STMA, the Shanghai Tobacco Monopoly Bureau (TMB), is authorized to design, implement, and enforce zoning regulations regarding tobacco retail under the national guidelines. In China, and Shanghai in particular, various tobacco control policies similar to those of HICs have been introduced in recent years, including smoke-free environments at Expo 2010, which was the first local-level legislation on tobacco control in China that prohibits smoking in 13 kinds of public spaces.^36^ Furthermore, interventions less common in HICs have also been introduced, including national-level legislation prohibiting tobacco sales around schools with the aim of protecting adolescents from earlier smoking initiation. In 2012, another spatial restriction on regulating tobacco retail was implemented by capping the number of new tobacco retail licenses that could be issued within a district.^37^ However, this capping policy was abolished in 2018.

Therefore, given the lack of work in this area in China, the aim of this paper is to investigate the socio-spatial patterning of tobacco retailing across the city area by examining the spatial disparities of neighborhood tobacco retail availability as well as the association between tobacco retail availability and neighborhood deprivation.

## Methods

### Study Setting

Shanghai is a megacity of China with an administrative area of 6340.5 km^2^ and a population of 24.2 million. The smoking prevalence among adults of Shanghai was 19.7% (2019), but with large disparities between males (37.4%) and females (0.8%).^38^ The study included all residential communities (akin to a residential neighborhood) (*n* = 5432) with a mean population of 5888. In the process of rapid urban transformation, due to the historical legacy of the metropolitan city, the level of urban development varied across different areas of Shanghai. The more urbanized areas of the city are characterized by greater economic activity and better access to local infrastructure than the less urbanized areas. Following previous literature in Shanghai,^39^ the city was stratified by urban center, urban area, and suburb, which were delineated on the basis of the road network (see Supplementary Appendix 1). Levels of urbanity decreased from urban center to urban area and to suburb.^40^ The total number of communities classified as urban center and urban areas were 958 and 1762, respectively; 2712 communities outside the Outer Ring Road were classified as suburb.

### Tobacco Retailer Data

Data on tobacco retailers were acquired by web-scraping Points-of-Interest (POIs) from Baidu Map, which is the biggest online map server in China. As a large and comprehensive collection,^41^ POIs have been used in previous research including work on amenities such as retailers.^42^ POIs cover three items of information including name, type, and coordinates. The process of web-scraping is based on keyword searching. Keywords including “convenience store”, “supermarket”, and “tobacco and alcohol outlet” were used to identify the three major types of tobacco retailers in the assessment of neighborhood-level exposure of tobacco retailing. Through “Ospider” (https://github.com/skytruine/OSpider), a total number of 106 889 POIs with duplications were acquired in May 2019.

The process of data validation and cleaning was completed in four steps (Supplementary Appendix 2). In Step 1, duplicates were removed, leaving a total of 20 126 POIs for further cleaning. The data were validated through daytime fieldwork in August 2019. Specifically, 470 POIs across the city were randomly sampled, located, and observed on foot. Through field validation, the following features could be applied to identify tobacco retailers in the online street view of Baidu Map for further validation in step 2: (1) an operating retailer may display a symbol of China National Tobacco Corporation (CNTC), and/or display products/advertisements inside or outside the retailer; (2) for convenience store and supermarket chains, one retailer may be confirmed as a tobacco retailer if any other chain store has been identified as a tobacco retailer (except for chain stores run by wholly foreign-owned enterprises); (3) all tobacco-only outlets were tobacco sellers.

In step 2, based on the features of an operating tobacco retailer, all POIs were verified through searching the images in the online street view of Baidu Map collected before 2019 using the name and address. A total of 19 362 POIs were retained as verified tobacco retailers after removing 4970 out of 25 126 POIs that were identified as not selling tobacco or had closed. A further 794 POIs could not be verified directly due to the coverage in street view, which required further validation. In step 3, further validation was performed to examine the unverified 794 POIs through the published official records of Shanghai TMB which contained information on 4652 legitimate tobacco retailers in 2014–2018. Through manual examination, 51 out of 794 POIs that were consistent with the official records could be confirmed as operating tobacco retailers. Finally, the invalidated POIs (*n* = 743, 3.69%) were excluded from the subsequent analysis, leaving a total of 19 413 verified tobacco retailers across Shanghai. By 3-level urban classification, 2680 tobacco retailers were distributed in the urban center while the total number of tobacco retailers was 5280 and 11 453 in the urban area and the suburb, respectively. The types of tobacco retailers retained for analyses were: (1) convenience stores (including convenience store chains, groceries or corner shops, gas stations, *n* = 9436); (2) supermarkets (*n* = 6319); and (3) tobacco-only outlets (including tobacco-only outlets and liquor stores that sell tobacco products, *n* = 3658). Considering that the marketing strategies and purchasing patterns of smokers may vary by different retailer types, only the three major types of tobacco retailers were retained for subsequent analyses.

### Neighborhood Deprivation

Following previous studies in China,^43,44^ four neighborhood-level indicators, including unemployment rate (unemployed residents), low-skilled workers rate (residents who were blue-collar or pink-collar workers), low-educational attainment rate (residents with junior high school education or below), and non-home ownership rate (residents who are tenants), were extracted from the 2010 national census of China at the residential community-level (the smallest administrative division in urban China) and combined to create a single deprivation measure. The four census-based indicators were standardized using Z-scores. Consistent with international indices such as the Carstairs and Townsend indices in the United Kingdom, all indicators were weighted equally and summed; a higher score indicates a higher level of neighborhood deprivation (see Supplementary Appendix 3).^45,46^ Further, all neighborhoods were grouped into quintiles of deprivation (Q1 = least deprived, Q5 = most deprived). Stratifying neighborhood deprivation quintiles by urbanity level, the suburb had a greater proportion of the more deprived neighborhoods (Q3–Q5), whereas the urban areas were found to have a greater proportion of less deprived neighborhoods (Q1–Q2) (see Supplementary Appendix 4).

### GIS Spatial Analysis

First, using ESRI ArcGIS 10.7.1 Geographical Information System (GIS), all validated tobacco retailers (*n* = 19 413) were mapped based on the coordinates of POIs within 5432 communities. Kernel density estimation (KDE) was selected to assess geographical availability because it transforms outlet locations into a continuous surface (across Shanghai) that is unconstrained by administrative boundaries. Thus, following methods used previously, the city of Shanghai was separated into 100 × 100 m grids, and the number and proximity of tobacco retailers with a radius of 800 m (a plausible 10-min walking distance) were calculated.^15,24^ An advantage of the KDE method is that it places greater weight on tobacco retailers near the center of the search window (the kernel) and less weight on tobacco retailers that are further away.^15^ The KDE value represents the smoothed density of tobacco retailers within the city of Shanghai (Supplementary Appendix 5).

The kernel density values for all tobacco retailers and each type of tobacco retailer were calculated and extracted to the centroids of their subcommunities. The total population of each subcommunity was acquired through the 2010 Chinese census of Shanghai. Neighborhood tobacco retail availability (TRA), defined by the density of tobacco retailers within a given neighborhood, was represented using the population-weighted KDE of all tobacco retailers for all centroids of subcommunities within each residential community:


$$\begin{array}{l} KDE_{i}=\frac{\overset{n}{\underset{j=1}{\sum }}(KDE_{j}\times pop_{j})}{\overset{n}{\underset{j=1}{\sum }}pop_{j}} \end{array}$$

where for each residential community i, $KDE_{i}$ is the population-weighted KDE value, n is the total number of subcommunities within each residential community, $KDE_{j}$and $pop_{j}$ are the density value and population of the *j*th subcommunity within community i. The TRA within neighborhoods presents a proximity estimate of the density of tobacco retailers (retailers per km^2^). Further, the distribution of population-weighted KDE values was tabulated and plotted to examine the distribution of neighborhood TRA across the city and by 3-level urbanity.

### Statistical Analyses

Descriptive statistics were used to characterize the spatial distribution of tobacco retail availability across the city of Shanghai by different types of tobacco retailers. Because of the non-normal distribution of the neighborhood TRA (skewness ranged from 0.49 to 2.64; kurtosis ranged from 2.97 to 10.28), median values were used to summarize the results. For each type of tobacco retailer, the median TRA was summarized across quintiles of neighborhood deprivation. The Kruskal–Wallis test, a non-parametric rank-based test (equivalent to a parametric one-way ANOVA), was performed to examine whether TRA differed by neighborhood deprivation quintiles statistically. Furthermore, the effect size was determined using epsilon squared to measure the strength of association between neighborhood deprivation quintiles and TRA.^47^ The value of epsilon squared ranges from 0 (no relationship) to 1 (perfect relationship).^47^ All analyses were stratified by the 3-level urbanity of the city to examine the spatial disparities.

All statistical analyses were conducted using the R packages “Tidyverse” and “Rcompanion”. “GGplot2” was used for plotting the results of descriptive statistics. ESRI ArcGIS 10.7.1 Geographical Information System (GIS) was used for spatial analyses.

## Results

### Spatial Distribution of Tobacco Retail Availability

The overall median TRA for all tobacco retailers was 9.50 tobacco retailers per km^2^ per neighborhood (95% CI 9.13–9.87). Decreasing with levels of urbanity, the TRA was highest in neighborhoods classified as “urban centre” (20.18 95% CI 19.62–20.94) and lowest in “suburb” neighborhoods (3.10 95% CI 2.87–3.40) (Table 1).

**Table 1.** Distribution of Population-weighted KDE of Tobacco Retailers

Median population-weighted KDE of neighborhoods (95% CI)				
Type of tobacco retail	Urban center	Urban area	Suburb	Overall
All	20.18 (19.62, 20.94)	12.69 (12.20, 12.96)	3.10 (2.87, 3.40)	9.50 (9.13, 9.87)
Convenience store	10.86 (10.57, 11.38)	5.69 (5.48, 5.89)	1.20 (1.11, 1.33)	4.18 (3.96, 4.42)
Supermarket	3.51 (3.35, 3.66)	3.14 (3.04, 3.24)	1.13 (1.05, 1.22)	2.37 (2.28, 2.46)
Tobacco-only outlet	4.98 (4.69, 5.33)	2.85 (2.66, 3.00)	0.15 (0.11, 0.22)	1.65 (1.53, 1.74).

### Neighborhood Deprivation and Tobacco Retail Availability

**Figure 1.**
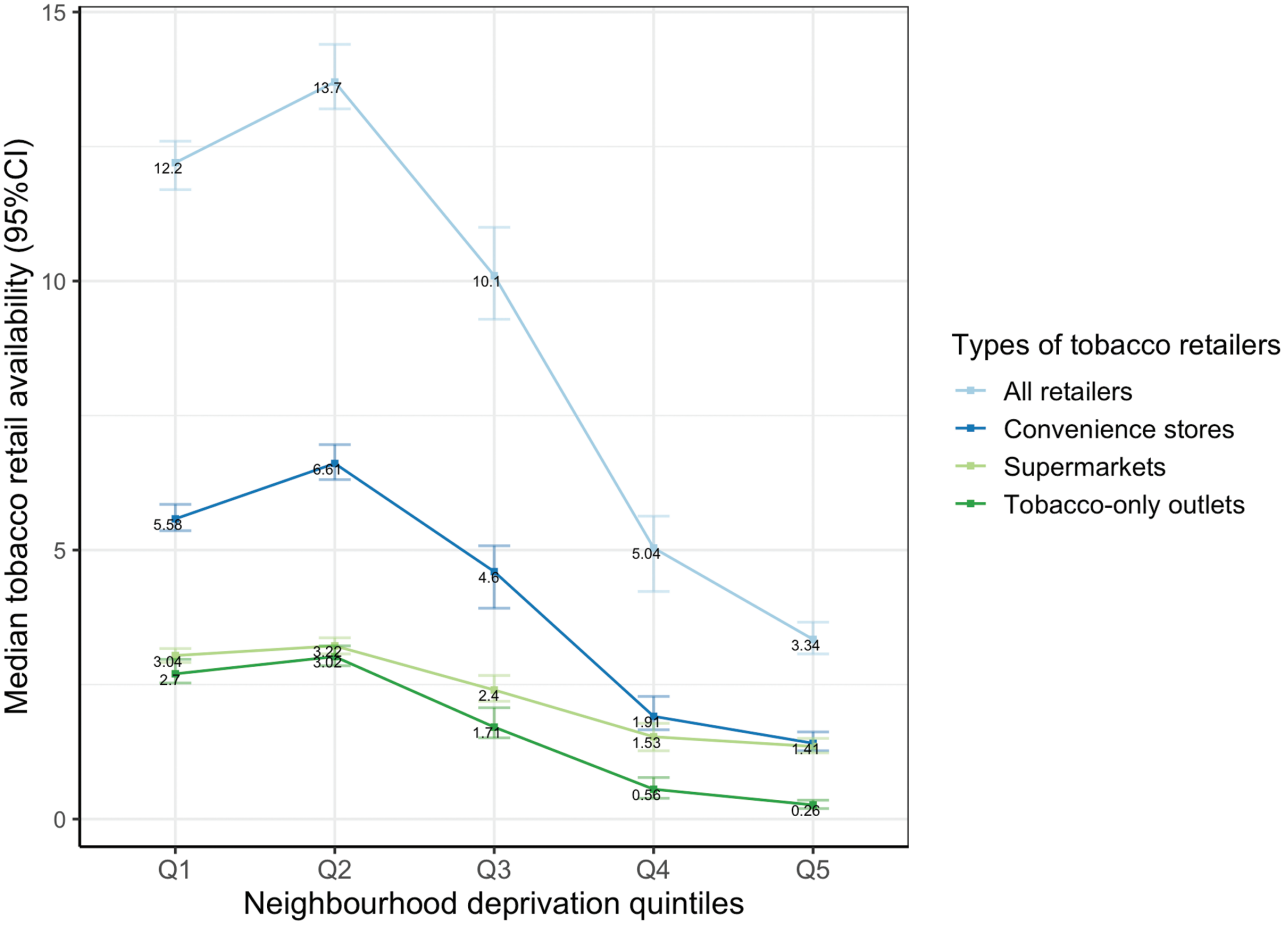
The overall association between neighborhood deprivation quintiles and tobacco retail availability.

Stratifying the median TRA by neighborhood deprivation demonstrated the profile was non-linear; with the TRA rising from the least deprived quintile (Q1), peaking in the less deprived quintile (Q2), and then dropping to a very low level (Figure 1) in quintile 5 (most deprived).

**Figure 2.**
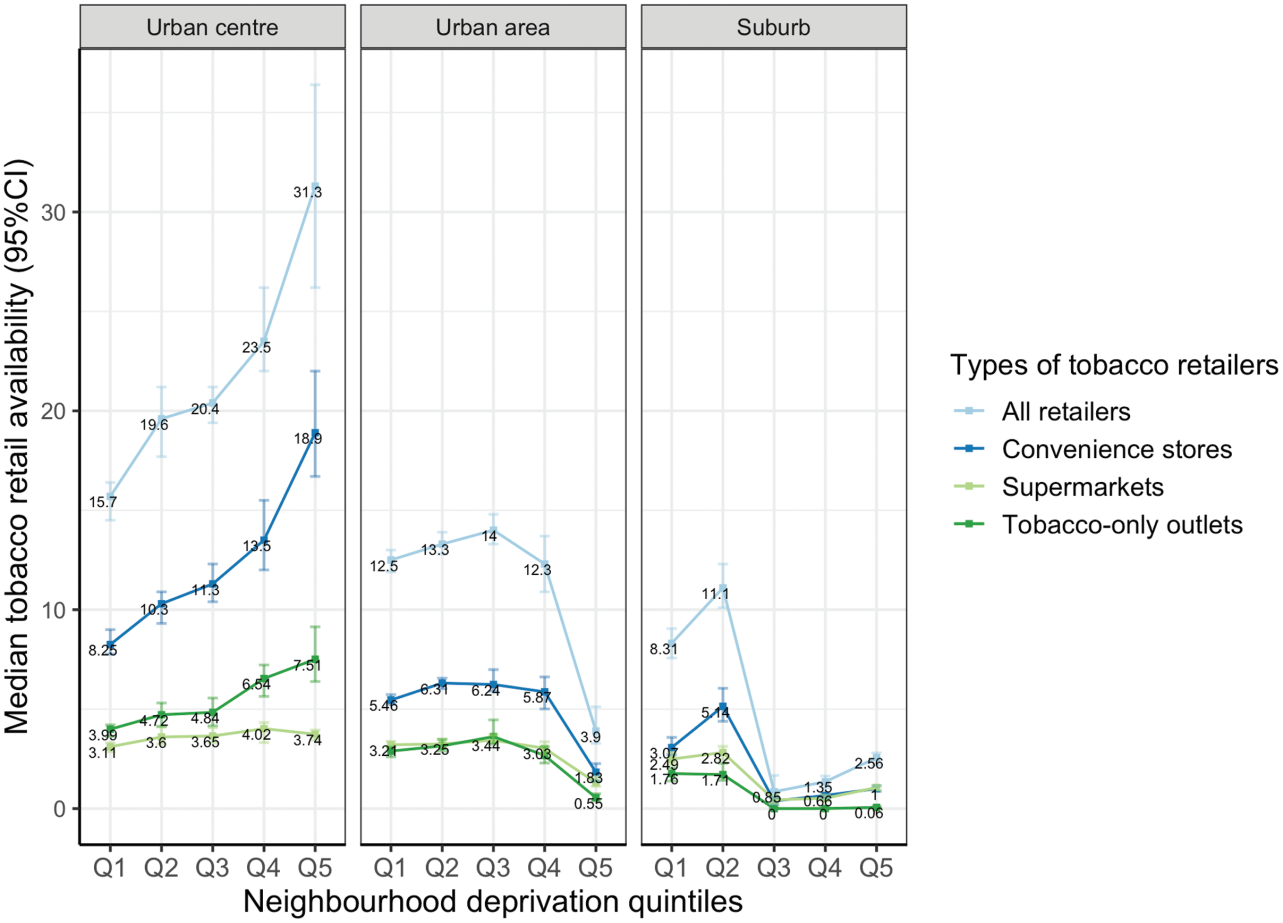
The association between neighborhood deprivation quintiles and tobacco retail availability by 3-level urban classification.

The association between neighborhood deprivation and median TRA varied across urban center, urban area, and suburb neighborhoods (Figure 2). In urban center neighborhoods, there is a broadly linear increase from 15.67 to 31.29 in the median TRA between Q1 and Q5. The availability peaked in Q3 in urban area, whilst it peaked in Q2 in suburb. The lowest median TRA was found in Q5 for neighborhoods characterized as urban area, whereas for suburb neighborhoods, the lowest availability was found in Q3 with a slight increase in Q3–Q5 from 0.85 to 2.56.

The TRA of all tobacco retailers statistically differed by neighborhood deprivation quintiles (*p* < .001) (Table 2). Measured by epsilon squared, the effect size of neighborhood deprivation quintiles on TRA varied by 3-level urban classification. The association between neighborhood deprivation quintiles and TRA of all tobacco retailers was strongest in urban center neighborhoods (ε^2^ = 0.18), whilst the association was weaker in urban area neighborhoods (ε^2^ = 0.10) than in suburb neighborhoods (ε^2^ = 0.15).

**Table 2.** Median Population-weighted KDE by Types of Tobacco Retailers and Neighbourhood Deprivation Quintiles by Across the City, Urban center, and Suburb

	Neighborhood deprivation quintiles					Kruskal–Wallis chi-squared	Effect size ($\varepsilon^{2})$
	Q1 (least deprived)	Q2	Q3	Q4	Q5 (most deprived)		
Across the city							
All retailers	12.19	13.67	10.15	5.04	3.34	611.81	0.11
Convenience store	5.58	6.61	4.60	1.91	1.41	542.55	0.10
Supermarket	3.04	3.22	2.40	1.53	1.35	457.77	0.08
Tobacco-only outlet	2.70	3.02	1.71	0.55	0.26	668.61	0.12
Urban center							
All retailers	15.67	19.61	20.38	23.53	31.29	168.79	0.18
Convenience store	8.25	10.30	11.32	13.48	18.86	192.25	0.20
Supermarket	3.11	3.60	3.65	4.02	3.74	23.43	0.02
Tobacco-only outlet	3.99	4.72	4.84	6.54	7.51	102.59	0.11
Urban area							
All retailers	12.51	13.26	13.98	12.33	3.90	182.00	0.10
Convenience store	5.46	6.31	6.24	5.87	1.83	153.98	0.09
Supermarket	3.21	3.25	3.44	3.03	1.30	116.33	0.07
Tobacco-only outlet	2.89	3.15	3.62	2.67	0.55	175.90	0.10
Suburb							
All retailers	8.31	11.14	0.85	1.35	2.56	395.21	0.15
Convenience store	3.07	5.14	0.38	0.66	1.00	337.18	0.12
Supermarket	2.49	2.82	0.44	0.52	1.06	255.13	0.09
Tobacco-only outlet	1.76	1.71	0.00	0.00	0.06	387.73	0.14

All *p*-values < .001.

### Socio-spatial Disparities by Tobacco Retailer Type

In the final stage of the analyses, neighborhood TRA measures were calculated for each retailer type.

In Table 1, amongst the three types of tobacco retailers, the highest availability was found for convenience stores (4.18 95%CI 3.96–4.42) compared to supermarkets (2.37 95%CI 2.28–2.46) and tobacco-only outlets (1.65 95%CI 1.53–1.74). Across the 3-level urbanity, compared to tobacco-only outlets, the availability of supermarkets was greater in urban areas (3.14 95%CI 3.04–3.24) and suburb neighborhoods (1.13 95%CI 1.05–1.22). Within the urban center, the availability of convenience stores (10.86 95%CI 10.57–11.38) was 3 times as high as that of supermarkets (3.51 95%CI 3.35–3.66).

The association between neighborhood deprivation and TRA by types of tobacco retailers are presented in Table 2. The findings were broadly consistent with the pattern of overall TRA, with the highest availability in less deprived neighborhoods and the lowest availability in the most deprived neighborhoods. The results also show that, amongst three retailer types, the availability of convenience stores was highest across all levels of neighborhood deprivation and the lowest for tobacco-only outlets. On the other hand, the association between neighborhood deprivation and the availability of tobacco-only outlets was stronger (ε^2^ = 0.12) than that of convenience stores (ε^2^ = 0.10) and supermarkets (ε^2^ = 0.08).

Stratifying the results by urban classification showed that the availability of each retailer type across the three levels of urbanity was consistent with the overall trend, except for the availability of tobacco retail in supermarkets within urban center neighborhoods, where there was a slight decrease in Q5. The availability of tobacco-only outlets had the strongest association with urban area neighborhoods (ε^2^ = 0.10) and suburb neighborhoods (ε^2^ = 0.14). However, in urban center neighborhoods, the availability of convenience stores was more strongly associated with neighborhood deprivation (ε^2^ = 0.20) than supermarkets (ε^2^ = 0.02) and tobacco-only outlets (ε^2^ = 0.11).

## Discussion

### Summary of Findings

This study examined the socio-spatial patterning of tobacco retailers across Shanghai, China using cross-sectional web-based POIs and census data. As anticipated, the availability of tobacco retailers decreased from neighborhoods characterized as urban center to urban area to suburb. Further, there was a non-linear association between neighborhood deprivation quintiles and tobacco retail availability, which varied by levels of urbanity. Amongst the three major types of tobacco retailers, convenience stores were the most frequent type of tobacco retailers while the availability of tobacco-only outlets was more associated with neighborhood deprivation quintiles.

### Interpretations of Key Findings

The significant negative association between neighborhood deprivation and tobacco retail availability is inconsistent with previous findings in HIC countries, where tobacco retailers tend to be more available in more deprived neighbourhoods.^20,21,23^In contrast, in Shanghai, the most deprived neighborhoods had the lowest availability of tobacco retailers, suggesting that residents of the most deprived neighborhoods are disadvantaged in their access to a range of basic resources, including various types of retailers.^24^ In addition, the lowest availability of tobacco retailing in the most deprived neighborhoods may be partly a consequence of low purchasing power among residents of these areas,^24^ which is consistent with the previous evidence in China suggesting that lower-income households spend less on tobacco products^48^; smokers from the most deprived neighborhoods of Shanghai may purchase cheaper cigarettes from fewer retailers.

The finding that the highest availability of tobacco retailers was in the urban center is broadly consistent with work in North America^19,21^ and Scotland.^15,24^ Uneven urban economic development across Shanghai is a possible explanation for this patterning. Similar to all types of retailing, tobacco retailers tend to concentrate in the urban center. The retail market in suburban neighborhoods, which has seen a transformation from an agrarian to an industrialized economy in recent years, is still under development. By contrast, the retail market in the urban center of Shanghai is well-established and highly networked, with more commercial activity. Uneven consumer demand across the city may also help to explain the geographical differences in tobacco retailing, with more demand (ie more smokers) in parts of the city with a greater population density.^27^ In Shanghai, the population density in the urban center was five times that in the suburb (see Supplementary Appendix VI). From the perspective of policymaking, the findings could also be attributed to the activities of the local TMB,^28^ who issue licenses, oversee tobacco control in the city, and at the same time promote tobacco sales at the local level.^28,34^ In the former zoning regulation regarding tobacco retail in Shanghai (2012), to meet consumer demand, TMB permits higher caps on the thresholds for newly issued tobacco retail licenses in areas with more economic activity (ie more urbanized areas).^37^ The dual roles of TMB may intensify the geographical differences in tobacco retail availability.

The study findings also demonstrated that the relationship between tobacco retail availability and neighborhood deprivation varied across urban center, urban area, and suburb. In urban center neighborhoods, similar to findings from HICs,^21–23^ greater availability was found in more deprived neighborhoods. However, in suburban neighborhoods, there was a non-linear association between deprivation and the availability of tobacco retailing with the highest availability in deprivation quintile 2, and the lowest in quintile 3. It is feasible that this finding may be specific to large cities such as Shanghai whereby there is an ongoing transition in the suburban tobacco retail market driven by the rural-to-urban transformation in the suburbs.

The local TMB/tobacco industry may play an important role in the socio-spatial patterning of tobacco retail by different retailer types by preferring convenience store chains and tobacco-only outlets over other retailer types when issuing tobacco retail licenses. The convenience store chains provide a unified and established retail network with a widespread availability of point of sales across the city, which could benefit the development of a more efficient tobacco retail network,^49^ further ensuring the ubiquity and easy access to tobacco products. Most of the tobacco-only outlets are run by local TMB/tobacco corporations, which advantages tobacco-only outlets in terms of tobacco sales, promotion, and marketing.^49^ Further, albeit the trend remained non-linear, our study found the availability of tobacco-only outlets was more sensitive to the effect of neighborhood deprivation in contrast to convenience stores and supermarkets, which may be because the tobacco-only outlets seek to maximize profits by targeting consumers from less deprived backgrounds with better purchasing power. The finding emphasizes the conflicting roles of local TMB, suggesting an urgent need for a greater separation between tobacco control policymaking and the tobacco industry.

### Limitations

The study has limitations. First, due to the lack of a comprehensive tobacco retailer register, the completeness of POIs regarding tobacco retailers cannot be assessed formally. Further, the POIs data do not include information regarding the size of tobacco retailers, the volume of sales, and the sub-types of major tobacco retailer types, which could be used to better characterize tobacco retail availability across the city. Further, since illicit tobacco retailers were not identifiable from the POI database, our study was restricted to legitimate tobacco retailers. Unlike earlier work,^50^ our study also did not include tobacco “kiosks”. However, the number of kiosks across the cities of China has decreased dramatically since 2012,^51^ and by 2019, the number of kiosks in Shanghai was estimated to be fewer than 300.^52^ Although the Shanghai TMB hold data on all legitimate tobacco retailers across Shanghai, the data are not available to researchers. Therefore, POIs provide the most comprehensive open-source data relating to tobacco retailing. Second, the study relies on cross-sectional data where measurements, including the TRA constructed by POIs and the census-based neighborhood deprivation index that were constructed at different timepoints using the most recently available data (2019 and 2010 respectively). However, between 2010 and 2019, a large amount of cultivated land around the urban periphery of Shanghai has been developed for residential and commercial purposes. These areas may have experienced socioeconomic changes that were not considered. Finally, this research focuses on socio-spatial patterning of exposure to tobacco retail and does not consider impacts on smoking (eg smoking rates, daily smoking) or health outcomes. Further research in China linking geospatial tobacco retail to smoking behavior data is urgently needed to investigate the relationship between neighborhood deprivation, tobacco retail availability, and individual-level smoking.

## Conclusion

The findings in this paper suggest that in Shanghai there was a greater availability of tobacco retailing in more urbanized areas and medium-deprived neighborhoods, with convenience stores being the most common type of tobacco retailer across the city. Examining the socio-spatial patterning of tobacco retail could provide crucial evidence for further tobacco control policies in Shanghai and other megacities of China.

As a signatory of the WHO Framework Convention on Tobacco Control (FCTC), China has made some progress in the development of tobacco control initiatives, particularly over the past six years, including smoke-free ordinances, cessation support, health warnings on tobacco products, and a ban on tobacco advertising and sponsorship.^3^ However, there is still a long way to go for tobacco control in China, not least the full implementation of the WHO FCTC, and to achieve the ambitious goals of reducing smoking prevalence and health inequality detailed in Healthy China 2030,^53^ a broad range of additional measures need to be considered. These measures require a comprehensive tobacco control strategy, including addressing the ubiquitous local availability of tobacco products in China.

From the perspective of reducing socioeconomic inequalities in smoking and improving the effectiveness of tobacco control strategies, this study emphasizes the need for greater efforts in regulating tobacco retailing at the neighborhood level. In addition to reducing the overall availability of tobacco retailers, policy makers should consider the possibility of implementing neighborhood-level spatial restrictions on different types of tobacco retailers, for example, through changes to the tobacco retail licensing process. Policy interventions in this area should also be cognizant of the significant socio-spatial disparities between different types of tobacco retailers as well as the advertising, promoting, and marketing activities among different types of tobacco retailers.^54^ Our study provides important evidence that place-based spatial restrictions that aim at reducing neighborhood-level tobacco exposure should form a key part of further policy interventions to address the social and geographical differences in smoking.

## Supplementary Material

A Contributorship Form detailing each author’s specific involvement with this content, as well as any supplementary data, are available online at https://academic.oup.com/ntr.

ntac155_suppl_Supplementary_Material

ntac155_suppl_Supplementary_Taxonomy-form

## Data Availability

Data are available upon reasonable request.
