# Supplementary material for: A Cross-sectional Analysis of Socio-spatial Patterning of Tobacco Retail in Shanghai, China
Source: Nicotine Tob Res. 2022 Jul 2;24(12):2018–25. doi: 10.1093/ntr/ntac155 (PMC9653074; doi:10.1093/ntr/ntac155)
Supplement: ntac155_suppl_Supplementary_Material [file ntac155_suppl_supplementary_material.pdf]

SUPPLEMENTARY MATERIALS

Appendix I. 3-level urban classification of Shanghai

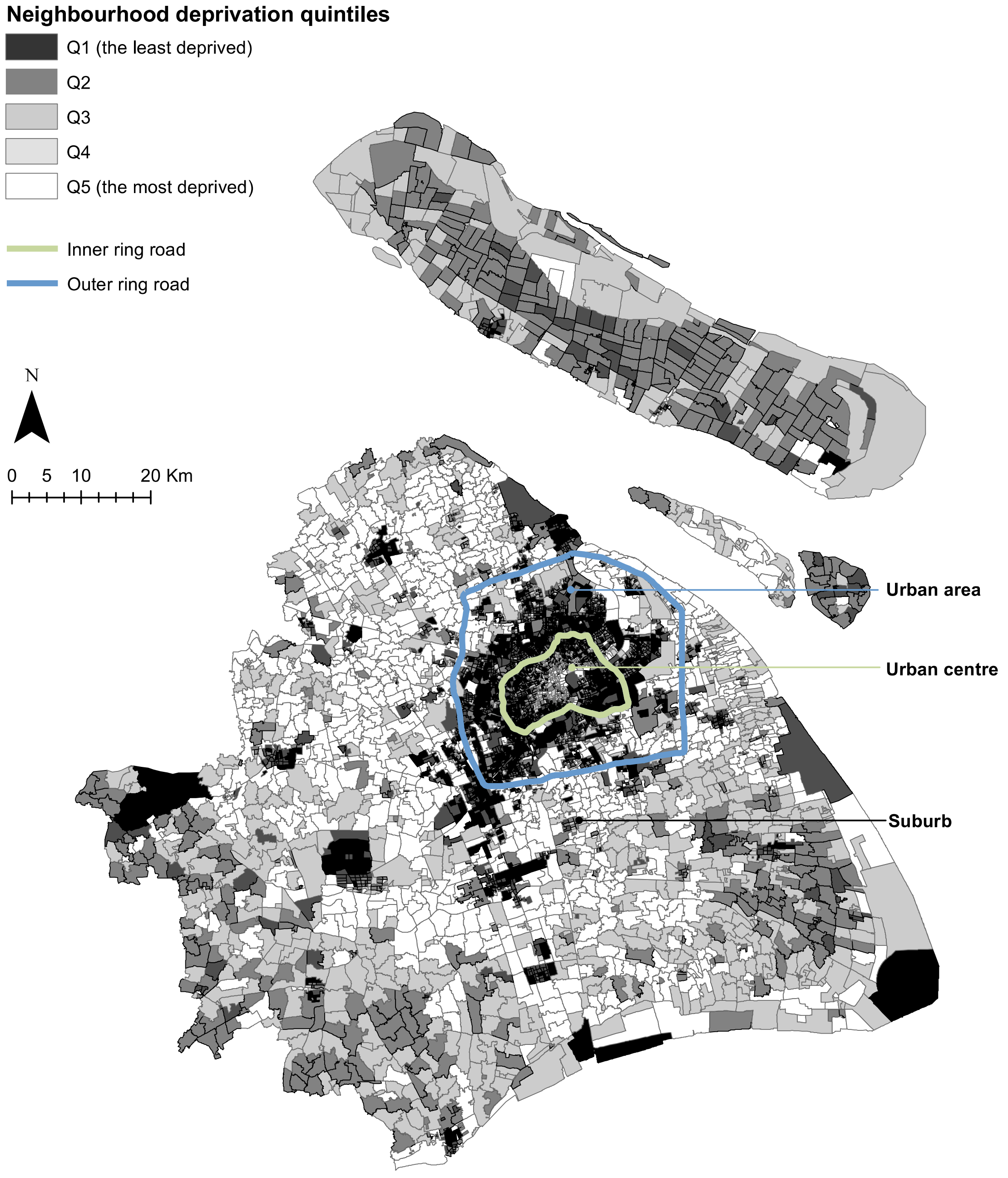

Appendix II. Workflow of tobacco retailer data validation and cleaning

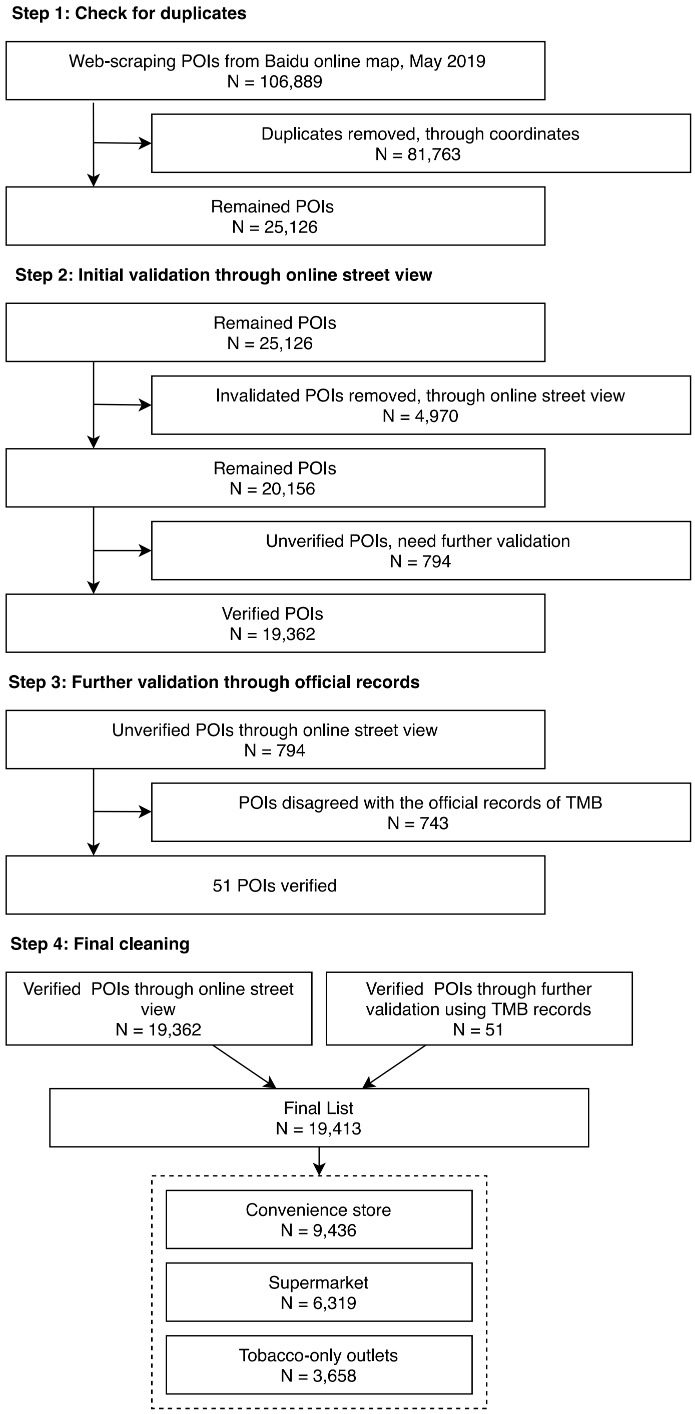

Appendix III. Descriptive statistics of deprivation index based on the 6th census of China using equal-weighting approach

Indicators	Definition	Mean (SD) %							
		Urban centre		Urban area		Suburb		Overall	
Unemployment rate (%)	Percentage of unemployed residents.	9.96	(5.18)	9.68	(5.90)	4.48	(4.88)	7.13	(5.91)
Low-skilled workers rate (%)	Proportion of residents who are blue-collar worker and pink-collar worker.	23.80	(10.44)	26.22	(14.10)	46.17	(16.99)	35.75	(18.35)
Education of junior school or below (%)	Proportion of residents with education of junior school or below.	38.41	(13.25)	41.41	(15.08)	67.29	(18.48)	53.80	(21.39)
Non-homeownership (%)	Proportion of residents who are tenants.	44.75	(28.32)	29. 49	(21.06)	36.80	(27.21)	35.83	(26.12)

Appendix IV. Distribution of neighbourhood deprivation quintiles, N(%)

**No. of residential communities (%)**					
**Urban classification**	**Q1**	**Q2**	**Q3**	**Q4**	**Q5**
Urban centre	277 (25.48)	203 (18.69)	174 (16.01)	146 (13.44)	158 (14.55)
Urban area	533 (49.03)	51 (47.42)	333 (30.63)	220 (20.26)	161 (14.83)
Suburb	277 (25.48)	368 (33.89)	580 (53.36)	720 (66.30)	767 (70.63)
**Overall**	1087 (100.00)	1086 (100.00)	1087 (100.00)	1086 (100.00)	1086 (100.00)

Appendix V. Kernel density estimation of tobacco retailers in ArcGIS 10.7.1

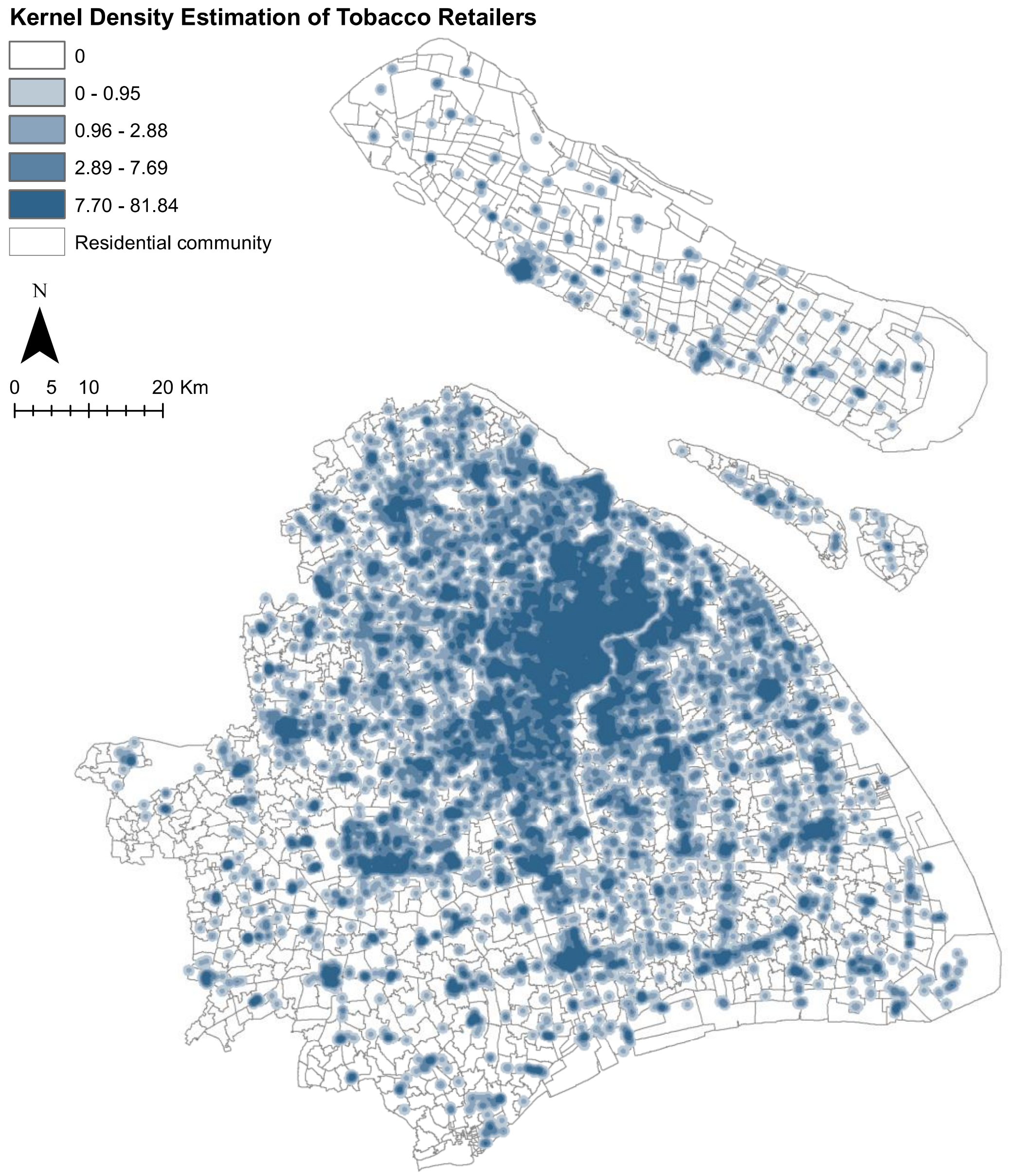

Appendix VI. Spatial distribution of neighbourhood population count, population density, count of subcommunities by means

	Population count (SD)	Population Density (SD) (per km^2)	No. of subcommunities within neighbourhoods (SD)
**Overall**	5888.17 (5020.95)	23406.12 (23773.40)	15.37 (9.76)
**Urban centre**	4539.27 (2029.80)	45972.97 (27563.41)	12.77 (6.11)
**Urban area**	5969.74 (4216.86)	33710.08 (20232.88)	16.29 (9.28)
**Suburb**	6311.67 (6055.72)	8739.97 (11574.69)	15.69 (10.90)

Appendix VII. Definitions on retailer types, count (%) of tobacco retailers by types

Retailer type	Definitions	N (%)
Convenience store	Convenience store chains, corner shops, gas stations, groceries	9,436 (48.61)
Supermarket	Mega, small and medium supermarket (chains)	6,319 (32.55)
Tobacco-only outlet	Tobacco specialty, liquor store that selling tobacco products	3,658 (18.84)
Overall	--	19,413 (100)
